# Is Misoprostol Vaginal Insert Safe for the Induction of Labor in High-Risk Pregnancy Obese Women?

**DOI:** 10.3390/healthcare9040464

**Published:** 2021-04-14

**Authors:** Valentin Nicolae Varlas, Georgiana Bostan, Bogdana Adriana Nasui, Nicolae Bacalbasa, Anca Lucia Pop

**Affiliations:** 1 Department of Obstetrics and Gynecology, Filantropia Clinical Hospital, 011132 Bucharest, Romania; 2 Department of Obstetrics and Gynecology, Carol Davila University of Medicine and Pharmacy, 37 Dionisie Lupu Street, 020021 Bucharest, Romania; nicolaebacalbasa@gmail.com; 3 County Emergency Hospital St. John the New, 720224 Suceava, Romania; bostangeorgiana@yahoo.com; 4 Department of Community Health, Iuliu Hațieganu University of Medicine and Pharmacy, 6 Louis Pasteur Street, 400349 Cluj-Napoca, Romania; 5 Department of Clinical Laboratory, Food Safety, Carol Davila University of Medicine and Pharmacy, 6 Traian Vuia Street, 020945 Bucharest, Romania or anca.pop@umfcd.ro

**Keywords:** misoprostol vaginal insert, induction of labor, pregnancy, obesity

## Abstract

Induction of labor (IOL) is an event that occurs in up to 25% of pregnancies. In Europe, the misoprostol vaginal insert (MVI—Misodel^®^) was approved for labor induction in 2013. Studies on the outcomes and safety of IOL in obese pregnant women are scarce; no data are available on MVI IOL in high-risk pregnancy obese women (HRPO—late-term, hypertension, diabetes). As the obesity rates are growing steadily in pregnant women, we aimed to evaluate the failure rate for induction and the safety of a 200 μg MVI in obese (body mass index (BMI) >30 kg/m^2^) HRPO compared to that for obese non-high-risk pregnancies (non-HRPO). For this purpose, we conducted a cross-sectional study in “Filantropia” Clinical Hospital, Bucharest, Romania, from June 2017—the date of the initiation of the MVI IOL protocol in our clinic—to September 2019. The primary outcomes were the failure rate, measured by cesarean section (CS) ratio, and secondarily, the safety profile of MVI, analyzed by one-way ANOVA. Out of a total of 11,096 registered live births, IOL was performed on 206 obese patients. Of these, 74 obese pregnant women had their labor induced with MVI (HRPO, *n* = 57, and non-HRPO, *n* = 17). The average maternal age was 29.9 ± 4.8 years (19–44 years). Across the groups, the rate of CS was 29.8% (*n* = 17) in the HRPO group compared to 23.5% (*n* = 4) in the non-HRPO group (*p* = non significant). In the vaginally birth subgroups, the median time from drug administration to delivery was shorter in the HRPO group compared to the non-HRPO group (16.9 ± 6.0 h 95% confidence interval (CI) 15.0–18.8 vs. 19.4 ± 9.2 h 95% CI 13.8–25.0, *p* = 0.03). No significant differences were found regarding the maternal outcomes among the studied groups; in terms of perinatal outcomes of safety, 5.4% (*n* = 4) of the cases of vaginal delivery for HRPO were associated with neonatal intensive care unit (NICU) admissions. The MVI seems to be an efficient labor induction agent in high-risk pregnancy obese women with good maternal outcomes and low perinatologic complications.

## 1. Introduction

The induction of labor (IOL—artificially initiated labor) is an event that occurs in up to 25% of pregnancies [1,2]. In Europe, the overall rates of IOL are between 7% and 33.0% [3,4]. Cervical status is a good predictor of vaginal delivery and is evaluated using Bishop’s scoring system [5]. Any induction method is effective in a woman with a favorable cervix (Bishop’s score ≥6), but the likelihood of obtaining a vaginal delivery decreases in women with unfavorable cervices (posterior, firm, and long) [5,6,7].

Cervical ripening is a physical process that increases the softening and distensibility of the cervix, leading to cervical effacement and dilatation; this mechanism is governed by complex biochemical, hormonal, inflammatory, and vasodilatory changes [8]. The endogen prostaglandins, originating from the cervix, uterus, placenta, and fetal membranes, play a critical role in cervical ripening [8]. Whenever necessary, iatrogenic cervical ripening is obtained using mechanical agents (insertion of catheters, cervical dilators, amniotomy) or pharmacological agents (application of prostaglandins, oxytocin, and smooth muscle stimulants, such as herbs or castor oil) [1,5,6,9,10]. The main problems experienced during the induction of labor are ineffective labor and excessive uterine activity, which may cause fetal distress and lead to an increased risk of C-section [10].

Prostaglandins′ (PGs) effects in the complex inflammatory and immune responses are mediated through specific transmembrane receptors—G protein-coupled receptors (GPCR) [11]. The therapeutic use of natural PGs is limited due to their rapid metabolism, their complex physiological activity that generates numerous (side) effects, and their chemical instability, leading to a short shelf life [12]. Two different types of synthetic prostaglandins are used for the induction of labor: the synthetic analog of the natural prostaglandin E1 (PGE1), misoprostol (most commonly used), and a prostaglandin E2 (PGE2) analog, dinoprostone [13].

**Figure 1:**
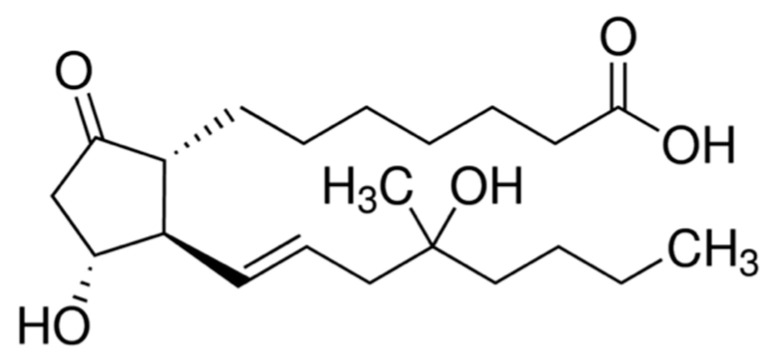
Misoprostol chemical structure. (±)-15-deoxy-(16RS)-16-hydroxy-16-methyl prostaglandin E1 [17].

Misoprostol is more effective for unfavorable cervix than other methods such as oxytocin, dinoprostone, and placebo, with no differences in adverse perinatal or maternal outcomes [9]. Until 2002, misoprostol was used off-label for cervical ripening and labor induction [14,15] in the uterotonics class G02AD [16]. In 2002, the Food Drug Agency (FDA) removed from the label misoprostol’s absolute contraindication in pregnancy [14]. Compared to other prostaglandin analogs, misoprostol is economical, widely available, stable at room temperature, and has few side effects [12,14]. Misoprostol’s chemical structure differs from PGE1 by a methyl ester group at C−1, a methyl group at C−16, and a hydroxyl group at C−16 rather than at C−15 (Figure 1). These minor differences increase the anti-secretory potency, improve oral activity, increase the duration of action, and improve the drug’s safety profile [12].

Misoprostol’s uterotonic and cervical-ripening actions are generally used in obstetrics and gynecology. More than 30 dosage regimens are described regarding this matter [18,19], with side effects being dose-related, usually transitory, and well-tolerated [20]. Clinical manifestations of toxicity include hypertonic uterine contractions, fetal distress and death, hyperthermia, rhabdomyolysis, hypoxemia, respiratory alkalosis, and metabolic acidosis. The toxic dosage in humans is unknown, and there is no specific antidote [20]. The use of oral or vaginal misoprostol for IOL is common in practice, and major limitations of these methods are the failure to predict the effects of misoprostol and the onset of the side-effects (diarrhea, nausea, excessive uterine activity, changes in fetal heart rate—FHR patterns), alongside the difficulties in managing them [15,21]. The side effects are dose-dependent and more familiar with oral misoprostol than intravaginal preparations [15]. A misoprostol vaginal insert (MVI) system was developed to resolve this limitation, comprising a non-biodegradable hydrogel polymer loaded with 200 μg of PGE1 analog.

An MVI allows the release of misoprostol continuously for 24 h (approximately 7 μg/hour) while the insert remains in place, thus providing the correct dosing and reducing the incidence of adverse events. The reservoir is rapidly and easily removed if needed [15,22]. The induction of labor with an MVI in a study conducted by Rayburn et al. revealed a 50% release of the drug by 12 h and 80% by 24 h [23]. A study by Tang et al. showed that pharmacokinetics of misoprostol are related to the administration route. After the sublingual use of a single dose of misoprostol, the time to peak concentration was significantly shorter, and the bioavailability was substantially higher than that obtained after vaginal use [12]. The plasma levels of misoprostol were sustained for a long time after vaginal administration (six hours vaginal route vs. four hours via sublingual route) [24].

The American College of Obstetricians and Gynecologists”(ACOG) Practice Bulletin (no.107/146, 2014) [25,26], the Society of Obstetricians and Gynaecologists of Canada (SOGC) Clinical Practice Guidelines (no. 296, 2013) [27], and the World Health organisation (WHO) Recommendations (2018) [2] indicate the use of misoprostol for IOL, while the National Institute for Health and Care Excellence (NICE) Clinical Guidelines (2008) [28] recommend using it only in clinical trials or demise fetus. The SOGC, WHO, and NICE guidelines recommend IOL in late-term pregnancies (41 + 0–42 + 0 weeks), while ACOG only recommends IOL in post-term pregnancies (42 + 0–42 + 6 weeks). In pregnancies complicated with pregestational or gestational diabetes, all the guidelines recommended the IOL [25,26,27,28] (Table 1).

Worldwide, obesity represents a complex multifactorial disease and an important epidemic health condition among women of reproductive age. The prevalence of obesity has been increasing progressively during the last 10 years at an alarming rate [29,30]. Obese pregnant women are more likely to experience pregnancy complications (early pregnancy loss, fetal malformations, premature birth, stillbirth, large for gestational age fetus) [31].

A large meta-analysis on over one million pregnancies [32] showed that 47% of pregnant women have gestational weight gain higher than that recommended in the guidelines [32], and 17.3% of pregnant women are obese, generating a potentially higher incidence of maternal and fetal complications [21]. Obesity is frequently associated with an increased rate of failure to induce labor, accompanied by an increased rate of cesarean sections, thus increasing the morbidity of the cases, affecting future birth and pregnancy outcomes [31]. Complications are more frequent when pregnant obese women have comorbidities (diabetes and hypertension) [31]. We encounter a particular obstetrical situation in isolated obesity, and an expectant attitude will determine the evolution towards a prolonged pregnancy, followed by a series of maternal-fetal risks. The different degrees of obesity expressed by the body mass index (BMI) are accompanied by a failure rate regarding the induction of labor directly proportional to an increased BMI value [33].

There is a lack of data in the literature about the outcomes and safety of IOL in obese pregnant women [34]. Several studies focused on the postnatal outcomes of obesity in mother and child, but less known about the clinical experience with MVI in obese patients [21,30,33]. There are no available clinical trials on misoprostol vaginal insert (MVI) induction of labor (IOL) in obese high-risk pregnancy patients in the literature. Therefore, the primary aim of the present study is to evaluate the rate of failure in the induction of labor with a 200 μg MVI in high-risk obese (BMI >30 kg/m^2^) pregnant (HRPO) women (i.e., late-term pregnancy, hypertension, or diabetes) compared to obese non-high-risk pregnant (non-HRPO) women. The secondary aim is to evaluate the safety profile of MVI related to the mother and newborn outcomes in both groups.

## 2. Materials and Methods

We developed a cross-sectional clinical study in order to evaluate: the rate of failure of the induction of labor with a 200 μg MVI in high-risk obese pregnant women compared to that for non-high-risk obese pregnant women; the safety profile of MVI related to the mother and newborn outcomes in high-risk pregnancy obese patients over a period of 28 months.

We used Quetelet’s index (BMI) to classify obese pregnant women as class I—between 30 and 34.9 kg/m^2^, defined as moderately obese; class II—between 35 and 39.9 kg/m^2^, defined as severely obese; and class III—over 40 kg/m^2^, defined as very severely obese. At admission to the hospital for IOL, weight (kg) was measured with a calibrated weight scale and height (m) with a calibrated stadiometer. Body mass index was calculated as weight (kg)/height squared (m^2^).

### 2.1. Study Design

We performed a retrospective cross-sectional study on obese pregnant women who had their labor induced vaginally with misoprostol. The subjects were recruited for a period of 28 months (from June 2017 to September 2019). During the study period, prostaglandin labor induction was introduced for the first time in our obstetrics department’s labor induction protocols.

The study was performed in the “Filantropia” Clinical Hospital, University of Medicine and Pharmacy “Carol Davila” one of the largest maternity wards in the country, situated in Bucharest, Romania, a city with more than 2.5 million inhabitants. Our clinic is a tertiary obstetrical unit specialized in managing high-risk pregnancies, with around 5000 deliveries annually.

IOL was indicated for various maternal and fetal conditions according to the clinic’s standard practice protocols. We collected and analyzed data from 74 obese patients that performed MVI IOL according to our clinic protocol.

The inclusion criteria into the study were: alive singleton pregnancy, cephalic presentation, a gestational age of 37 completed weeks and above (on-term pregnancies or late-term, defined as delivery at 41–41 + 6 weeks of gestation), a parity less than three, and obesity, with BMI >30 kg/m^2^, with or without associated high-risk factors: hypertensive disorders (preeclampsia, gestational hypertension, and chronic hypertension) and diabetes (gestational diabetes mellitus (DM), pre-conceptional controlled insulin-dependent or noninsulin-dependent DM).

**Figure 2:**
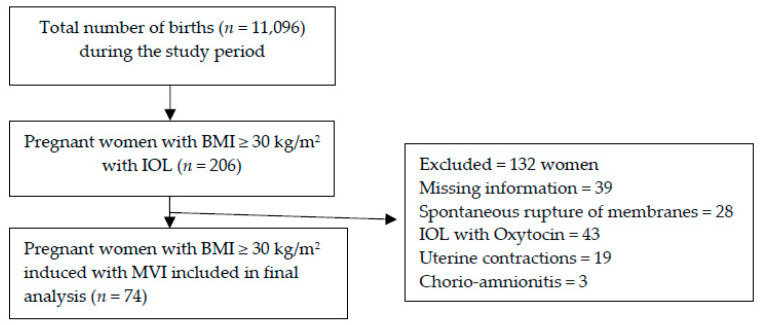
Flow-diagram of patient selection and distribution.

The exclusion criteria were parity of more than three; previous cesarean section; signs of fetal distress; antepartum hemorrhage; pre-labor rupture of membrane (PROM); BMI <30 kg/m^2^; severe preeclampsia or hemolysis, elevated liver enzymes, low platelet syndrome (HELLP); significant cardiovascular, renal, or hepatic disease; and complicated diabetes (nephropathy, retinopathy, neuropathy, or arteriopathy). The patient selection and distribution are depicted in the following diagram (Figure 2).

### 2.2. Clinical Evaluation and Data Collection

Before starting the procedure, the ultrasound evaluation of the amniotic fluid was performed, assessing the fetal presentation, fetal status, and weight. The gestational age was established by the correlation between the first-trimester ultrasound scan and the last menstrual period’s first day (LMP). Each patient underwent at least 20 min of cardiotocography assessment to ensure fetal status and evaluate the uterine contraction pattern for signs indicative of active labor. A clinical examination was performed to determine the baseline Bishop’s score. The reason for labor induction was recorded in the medical file.

Labor induction was guided using a standardized protocol for the procedure, using the vaginal insert system consisting of a controlled-release, retrievable polymer for the gradual delivery of 200 micrograms of misoprostol over 24 h, placed in the posterior vaginal fornix. Patients were monitored for uterine activity and fetal heart rate activity for at least 30 min after administering prostaglandins and throughout the entire labor, except for short periods (the need for toileting or ambulation).

The other parameters monitored to analyze the drug’s safety profile were: cervical status, time from induction to delivery, drug side effects, mode of delivery, and neonatal outcomes. Safety and efficacy analysis of the drug was done following maternal and neonatal outcomes. After drug insertion, vaginal examinations were performed every 4 h until 24 h if the delivery had not occurred, the Bishop score being recorded each time.

The time and mode of delivery of the neonate and instrumental vaginal delivery or C-section were recorded. The vaginal insert was removed at the onset of active labor (defined as ≥three contractions in 10 min, lasting 45 s, cervical change reaching 4 cm dilatation); after the 24 h dosing period; or at the occurrence of any intrapartum adverse event. If the membranes spontaneously ruptured, the vaginal insert was removed, and antibiotic prophylaxis was started after 12 h or immediately if a vaginal group B streptococcal smear test was positive.

Uterine tachysystole was defined as more than five contractions in 10 min over three consecutive 10 min periods, and in this situation, the tocolysis was initiated to control uterine activity. The initiation of the C-section protocol defined unsuccessful IOL.

### 2.3. Statistics

Data analysis was performed by using the SPSS^®^ 27.0 software (IBM^®^, Armonk, NY, USA). We used the Kolmogorov–Smirnov to assess the normal data distribution for all the variables. We described the continuous variables using the median (range), mean and standard deviation (SD) with a 95% confidence interval (CI) or count (percent, %), when appropriate. To compare proportions and the interdependence of nominal (categorical) variables, we used the chi-square test (χ^2^), applied in frequency comparisons. We performed the analysis of variance (ANOVA) test for continuous data to establish the influence of high-risk subgroups on continuous data (Bishop score, fetal weight, gestational age). In these cases, one-way ANOVA was performed. Results with *p* < 0.05 were considered statistically significant.

## 3. Results

During the study period of 28 months, out of the total 11,096 registered live births, 206 pregnant and obese patients were initially screened for eligibility for our study. The period prevalence of obese pregnant-labor induced was 186/10,000 births. One hundred and thirty-two cases were excluded due to exclusion criteria. Out of the total obese MVI-induced births, 35.9% (*n* = 74) of the patients fitted the inclusion criteria (Figure 2). Out of all of the obese women admitted to the clinic during the studied period and matching the inclusion criteria, 77% of the obese pregnant women were at high risk (HRPO), and 23% were non-high-risk (non HRPO). The participants′ average maternal age (mean ± SD) was 29.9 ± 4.8 years (19–44 years).

### 3.1. Description of the Studied Group

**Figure 3:**
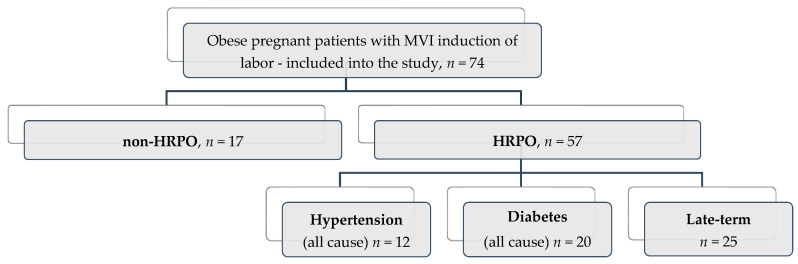
Distribution of the study subjects based on associated criteria in obese pregnant patients with misoprostol vaginal insert (MVI) IOL according to Strengthening The Reporting of Observational Studies in Epidemiology (STROBE) guidelines.

The cases′ stratification was undertaken according to maternal age, area of residence, BMI class, parity, gestational age, Bishop score, birth weight, neonatal intensive care unit (NICU) admissions (Table 2). The distribution of patients was as follows:

(1) High risk (HRPO, *n* = 57) stratified into three subgroups: late-term pregnancies (41–41 + 6 weeks of pregnancy) (*n* = 25, 43.8%), diabetes (*n* = 20, 35.1%), and hypertension (*n* = 12, 21.1%);
(2) Non-high risk (non HRPO, *n* = 17)—no comorbidities and on term (Table 2, Figure 3).

### 3.2. Evaluation of Failure Rate for Induction of Labor (IOL) with Misoprostol Vaginal Insert (MVI). Incidence of Cesarean Section

The failure rate of the HRPO group was 29.8%, clinical higher than in the non-HRPO group (23.5%, *p* = 0.46, Table 3).

The overall rate of successful labor induction associated with a vaginal delivery was 71.6% (*n* = 53). The initial Bishop score was 1.8 ± 1.3 (95% CI, 1.54–2.13). The mean interval with effective MVI action was 11.5 ± 5.4 h (95% CI 10.21–12.74), and the mean interval from induction to delivery was 17.3 ± 6.3 h (95% CI 15.8–18.8).

From the total of 53 vaginal deliveries, 79.2% (*n* = 42) were spontaneous vaginal deliveries and 20.8% (*n* = 11) were instrumental vaginal deliveries.

From the entire group (*n* = 74), 21 (23.4%) women underwent cesareans. Out of the 21 cesareans, 52.4% (*n* = 11) were carried out due to the failure of induction (lack of uterine contraction or cervical ripening after 24 h), 14.3% (*n* = 3) due to ineffective labor, 14.3% (*n* = 3) were carried out due to fetal distress, and 19% (*n* = 4) due to cephalo-pelvic disproportion.

### 3.3. Safety Profile

The main aim regarding MVI IOL’s safety is to identify the factors that precipitate the C-section delivery reported to maternal and neonatal outcomes amongst the study groups.

Side effects of misoprostol were registered in 17.6% cases (*n* = 13); two cases of tachysystole, one case of uterine hyper-stimulation, four cases of abnormal fetal heart rate (FHR) pattern, and six cases of meconium passage, leading to three cases (4.1%) of emergency *C*-section to deliver the infant. We found a low rate of uterine hyper-stimulation, and there was no difference in neonatal morbidity between groups.

Unfavorable fetal outcomes, assessed by the NICU admissions and the initial one-minute Apgar score ≤ 7, were associated with vaginal deliveries in the high-risk obese pregnancies group (*n* = 4, representing 5.4% of the total deliveries, *p*—not significant), mainly due to respiratory distress syndrome.

However, most cases with side effects of misoprostol were managed conservatively with the extraction of the vaginal insert and intravenous tocolysis.

The majority of women in the study group delivered vaginally without any significant impact on their infants Apgar score, except for three emergency C-section cases.

No general maternal side effects (nausea, diarrhea, chills, and fever) were recorded during IOL in our study.

### 3.4. Correlations between Study Subgroups and Outcomes of the MVI IOL

The MVI IOL outcomes in the studied group by type of risk factor and type of delivery were recorded (Table 4 and Table 5).

According to parity or maternal age, there were no statistically significant differences in maternal and neonatal outcomes in the studied pool. Mother age under 35 years was significantly linked to prolonged pregnancy (*p* = 0.047). No statistical significance was found between maternal age and birth type or Bishop score.

We found a good correlation in vaginal delivery between initial Bishop score and the time of misoprostol action in late-term (*r* = 0.51, *p* < 0.03) and hypertension (*r* = 0.97, *p* < 0.003) pregnancy subgroups.

When analyzing the vaginal birth subgroup, time induction to delivery is significantly shorter in women with IOL for HRPO women compared with non HRPO (16.9 ± 6.0 h 95% CI 15.0–18.8 vs. 19.4 ± 9.2 h 95% CI 13.86–25.0, *p* = 0.03), and with an average initial Bishop score of 1.7 ± 1.4 95% CI 1.4–2.1 versus 2.2 ± 0.8 95% CI 1.8–2.7, *p* = 0.147). We found a positive correlation between initial Bishop score and spontaneous birth (*p* = 0.028) in studied groups.

There is a significant statistical correlation between the late-term pregnancy subgroup and misoprostol action (*p* < 0.001) and between the pregnancy term and Bishop score (*p* = 0.001). There was no statistically significant difference between sub-groups in Bishop scores at the time of admission, the number of subjects who required oxytocin therapy during labor, or the mean amount of oxytocin administered.

## 4. Discussion

There is a significant variation in the labor induction approach in the clinical practice guidelines, with no standardization between the guidelines of different protocols, partly explaining the induction failure rate variability. The variability becomes obvious when we report the induction for a particular type of pregnant woman that associates, e.g., obesity, comorbidities (hypertension and/or diabetes), or other obstetrical risk (e.g., prolonged pregnancy). In the literature, we identified either conflicting recommendations on condition-specific guidelines or a lack of data related to the topic (e.g., obesity).

Usually, elective induction of labor is not recommended before 41 weeks of gestation in nulliparous women with a low Bishop’s score. Observational studies comparing elective induction of labor with expectant management did not demonstrate elevated cesarean section rates in the induction group or increased maternal and neonatal complications [35]. Other studies have demonstrated that the induction of labor between 38 and 39 weeks (a) did not increase the cesarean delivery rate, (b) significantly decreased the incidence of large-for-gestational-age infants, macrosomia, and shoulder dystocia [5,6].

In high-risk pregnancies, the moment of labor induction or the cesarean section’s time is not well established, especially when the obesity factor occurs. Thus, MVI for IOL was indicated or scheduled at >41 weeks in prolonged pregnancy, while there were inconsistent recommendations in diabetes (37–39 weeks in case of maternal or fetal complications; 40–41 weeks in the absence of other indications), and it was scheduled between 37 and 39 weeks in hypertension [26,36,37].

In particular, in obese post-term pregnant women, prolonged labor and higher cesarean rates are associated with impaired uterine contractility [38]; in class II obese post-term pregnant women, there is a significant risk of hypertensive disease [34], while in on-term diabetic obese pregnant women there is a decreased risk of perinatal mortality.

The induction of labor in obese women is associated with a high risk for cesarean section and an increased rate of maternal and neonatal complications [39,40]. However, the use of the routine or elective induction of labor in obese patients in high-risk pregnancy does not increase maternal and neonatal morbidity (respiratory distress, admission in NICU, and neonatal mortality), with a lower risk than allowing the pregnancy to progress after 41 weeks in the case of prolonged pregnancy, or the case of complicated diabetes or hypertension.

The short time from induction to delivery in HRPO compared to the non-HRPO group emphasizes the need to actively manage high-risk obese cases—in order to decrease complications. The differences in attitude consist of active behavior after the suppression of the intravaginal device [41]. Decreasing the time to delivery in high-risk obese women has several advantages: reduced infection rates, reduced use of antibiotics and oxytocin, and lower maternal distress [42]. A shorter response rate between the studied groups regarding the action of the oxytocin infusion is determined by an augmentation of the expression of the oxytocin receptors directly related to the pregnant woman’s BMI value, according to the study of Garabedian. Another study showed that obese women undergoing induction of labor with misoprostol have a longer duration of induction to delivery, require more oxytocin to augment labor, more misoprostol doses, and have a higher rate of cesarean delivery, probably due to impaired uterine activity [43]. Studies on uterine contractility have shown that a sustained misoprostol level, rather than a high serum level, is required to develop regular uterine contractions [12].

As stated by the international (ACOG, WHO, SOGC, NICE) [25,26,27,28] and national (SOGR—Romanian Society of Obstetrics and Gynecology) [44] guidelines, misoprostol vaginal insert is recommended for IOL in pregnant women, proved to be a reliable and safe method to support cervical ripening. In Romania and in certain European Union (EU) states (i.e., Germany) since the end of 2019, MVI is no longer available.

Among several studies which analyze the failure rate of induction in obese women with different cervical ripening methods [21,30,34], we decided to evaluate the impact of maternal obesity on high-risk pregnancy induction with a single dose of vaginal misoprostol. The single dose of misoprostol appears to be an acceptable alternative to a multiple-dose regimen for cervical ripening in the induction of labor in multiparous women with an unripe cervix [7]. In our IOL protocol, the usage of misoprostol vaginal insert containing a dose of 200 μg represents a fast and reliable method to induce labor, associated with a higher rate of vaginal delivery within 24 h, a shorter hospital stay. Literature shows that the use of MVI increases uterine tachysystole incidence without the possibility of the event prediction by demographic or clinical factors, without an increased rate of C-section [31,39]. The present study showed good maternal and neonatal outcomes with 13 cases of side effects from a total of 74; in 10 cases out of 74 (13.5%), the extraction of the vaginal insert and i.v. tocolysis effectively controlled the uterine activity and normalized the fetal heart rate (as previously found by Bolla et al. [45]); three emergency C-sections were performed in 4.0% of the total obese women.

The failure rate in prostaglandin labor induction is related to numerous associated factors (patient age, gestational age, BMI, Bishop score, epidural anesthesia, oxytocic infusion) that may cumulatively alter the overall risk of failure. The risk of induction failure in obese pregnant women with prostaglandins may be increased due to modifications to the myometrial matrix, secondary to a decreased intensity of contraction [12,19].

The maternal BMI is correlated with the duration of induced labor and failure induction rate (C-section rate). The latest study by Glazer et al. reported that term induction of labor was associated with reduced cesarean delivery risk among women with obesity and with or without comorbidities [33]. We report a rate of 28.4% (*n* = 21) of cesareans among the entire study group induced with MVI. This rate is lower than the rate of (35%) reported by Beckwith et al. [46] and similar (29.8%) to that in Pevzner et al.’s study [47]. Recently, Rossi et al., in a predictive model among obese women, found an IOL failure rate of 24.9% [48], which is quite equal to our failure rate in the non-HRPO group (23.5%), and lower compared with that (26%) reported by Stephenson et al. [15].

According to previous research, we observed that identifying the correct moment of labor induction (after 38 weeks) among high-risk obese women does not determine a significantly increased risk of C-section rate compared with expectant management of pregnancy (up to 41 weeks). However, the C-section’s main indication was complex because it was not always easy to incriminate one responsible factor [36]. A shorter time of admission to delivery and a reduced C-section rate was correlated with increased maternal satisfaction with labor induction [49].

We identify two essential predictors of maternal and fetal outcomes—pre-conceptual BMI and gestational weight gain. Furthermore, the obstetrician recommends the amount of weight gain during pregnancy at the first visit according to the pre-pregnancy BMI. Thus, maternal obesity is associated with a lower Bishop score and increased IOL failure [50]. In women with an increased BMI (classes II and III), labor progresses more slowly due to an increased active labor duration [42,43]. Our study does not have any women with class III obesity for labor induction (just classes I and II) in accordance with epidemiological studies, which reveals a low incidence of class III obesity in our population [51]. For class III obese women with BMIs over 40 kg/m^2^, Carhall et al. reported an association between BMI and active labor duration [52].

Evaluating the possibility of IOL failure in high-risk obese groups is important—to allocate the resources, to manage the complications, and to counsel the at-risk group. Furthermore, we do not stipulate that an elective C-section should be recommended on at-risk obese women, but it’s mandatory to manage these cases in a specialized tertiary center. Our confidence is that this study may help to assess the risks of comorbidities or special obstetrical conditions (late-term pregnancy) in delivery planning of obese pregnant women. The current protocol uses dinoprostone, less active than misoprostol, as published literature has revealed [53]. Future research is needed to evaluate which cervical ripening method is more efficient and safe for IOL in HRPO women.

### 4.1. Limitations

Our study’s main limitation is the small number of obese women included; prospective future studies are needed to identify which predictive factors can be used to select the labor induction of the obese patients with increased obstetrical risk. Furthermore, we do not have direct information about the BMI at the first visit, nor preconceptual BMI in data collection from patients′ records, because some of the patients started their antenatal care after the first trimester. We did not have any obese pregnant women in class III according to BMI. Further prospective studies—capable of producing more robust evidence, such as randomized clinical trials—are necessary to confirm the present data’s validity.

### 4.2. Strengths

Our study is the first cross-sectional study investigating high-risk pregnancy–induction of labor outcomes with misoprostol vaginal inserts in obese on-term or late-term patients. Previous studies on the effect of maternal obesity on labor induction evaluated only the late-term pregnancies induced by multiple therapy protocols with misoprostol, by amniotomy, and oxytocin infusion [39]. Viteri [54] recently reported on nulliparous obese women with IOL, using the combination of a Foley balloon and misoprostol, resulting in similar C-section rates ripening with vaginal misoprostol alone.

## 5. Conclusions

The misoprostol vaginal insert system is an efficient and safe drug system for labor induction with no statistically negative impact on the maternal or fetal outcome when used in high-risk obese pregnant women with late-term, hypertensive, and diabetes pathology, with good perinatal outcomes. The close monitoring of both the mother and fetus represents the critical priority within the safety protocol and a favorable obstetrical outcome under this drug’s administration profile.

The MVI induction of labor in high-risk pregnancies in our study was a reasonable and safe management option for obese women. Obese pregnant women should be advised of possible complications during pregnancy or childbirth and should be referred to a high-risk pregnancy unit. It is mandatory to counsel these patients about the chances of vaginal delivery in cases of MVI for the induction of labor.

Aware of risks and advantages, IOL with MVI is an alternative worth informing obese pregnant women about concerning the chances of vaginal delivery, with earlier effective contraction, a short time from induction to delivery, and decreased C-section rate.

## Figures and Tables

**Table 1:** Recommended use of misoprostol for induction of labor (IOL) across the guidelines.

High-Risk Pregnancies	WHO (2018)	ACOG (2014)	SOGC (2013)	NICE (2008)
Late-term pregnancy	Yes	No	Yes	Yes
Diabetes	Yes	Yes	Yes	Yes
Preeclampsia	N/A	Yes	Yes	N/A

**Table 2:** Description of study participant group structure and characteristics.

Demographic Data	Non-High-Risk Pregnant Obese Women (Non HRPO) *N* = 17	High Risk Pregnant Obese Women (HRPO) (*N* = 57)			*p*-Value *
		Late-Term *n* = 25	Diabetes *n* = 20	Hypertension *n* = 12	
Maternal age, mean (SD)	28.5 ± 6.3	29.4 ± 3.1	32.2 ± 3.9	29.3 ± 5.4	0.173
Area of residence					
Urban (*n*)	12	20	15	9	0.919
Rural (*n*)	5	5	5	3	
BMI class, mean (SD)					
obese class I	13 (76.4%)	18 (31.6%)	14 (24.6%)	10 (17.5%)	
obese class II	4 (23.6%)	7 (12.3%)	6 (10.5%)	2 (3.5%)	
Mean BMI (SD)	33.1 ± 2.6	34.1 ± 2.8	33.7 ± 3.1	32.5 ± 1.4	0.144
Parity					
Primiparous (*n*)	17	23	19	9	0.096
Multiparous (*n*)	0	2	1	3	
Induction data					
GA at delivery (weeks), mean (SD)	39.3 ± 0.8	41 ± 0	38.3 ± 0.5	38.5 ± 0.7	<0.001
Bishop score, mean (SD)	2.2 ± 0.8	1.3 ± 1.4	1.7 ± 1.2	2.6 ± 1.0	0.003
Neonatal outcomes					
Birth weight (grams), mean (SD)	3205.8 ± 301.3	3450 ± 0.8	3417.5 ± 478.8	3250 ± 342.4	0.165
NICU admission, *n* (%)	2 (11.7%)	2 (3.5%)	3 (5.2%)	2 (3.5%)	0.853

* *p* < 0.05 was considered statistically significant; BMI—body mass index; NICU—neonatal intensive care unit; GA—gestational age.

**Table 3:** Distribution of the mode of delivery regarding studied groups.

	HRPO * (*n* = 57) (*n*/%)	non-HRPO * (*n* = 17) (*n*/%)	*p*-Value
Vaginal deliveries	40 (70.2%)	13 (72.4%)	0.39
C-sections	17 (29.8%)	4 (23.5%)	0.46

* HRPO = high-risk pregnant obese women, *p* < 0.05 was considered statistically significant.

**Table 4:** Outcomes of the MVI IOL in the studied group by type of risk factor.

	Non HRPO Women (*n* = 17, 23.0%)		HRPO Women (*n* = 57, 77.0%)				*p*-Value	
	Mean ± SD	95% CI	Late-Term (*n* = 25) Mean ± SD	Diabetes (*n* = 20) Mean ± SD	Preeclampsia (*n* = 12) Mean ± SD	Total (*n* = 57) Mean ± SD		95% CI
Misoprostol action (h)	12.4 ± 5.7	9.5–15.3	11.0 ± 5.9	10.8 ± 3.9	12.3 ± 6.6	11.2 ± 5.4	9.8–12.6	0.435
Time induction to delivery (h)	18.4 ± 8.5	14.1–22.8	16.7 ± 5.5	16.6 ± 4.7	17.3 ± 7.4	17.0 ± 5.6	15.5–18.5	0.041
Initial Bishop score	2.2 ± 0.8	1.8–2.7	1.2 ± 1.5	1.7 ± 1.2	2.7 ± 1.1	1.7 ± 1.4	1.4–2.1	0.147
1-min Appgar score	8.5 ± 0.9	8.0–9.0	8.5 ± 0.7	8.4 ± 1.2	8.4 ± 0.8	8.5 ± 0.9	8.2–8.7	0.990
5-min Appgar score	9.2 ± 0.8	8.8–9.6	9.1 ± 0.6	9 ± 0.6	9 ± 0.6	9.0 ± 0.6	8.7–9.2	0.372
Weight (grams)	3205.9 ± 434.4	2982.5–3429.2	3450 ± 301.4	3417 ± 478.8	3417.5 ± 478.8	3396.5 ± 381.8	3295.1–3497.8	0.084
Gestational age (weeks)	39.8 ± 0.9	39.4–40.3	41.3 ± 0.2	38.9 ± 0.6	38.9 ± 0.6	39.9 ± 1.3	39.5–40.3	0.901

h—hours, min—minute, *p* = 0.05.

**Table 5:** Outcomes of the MVI IOL in the studied groups (non-HRPO and HRPO) by type of delivery.

	HRPO Women		Non HRPO Women		*p*-Value
	Mean ± SD	95% CI	Mean ± SD	95% CI	
**Cesareans (*n* = 21)**	***n* = 17, 81.0%**		***n* = 4, 19.0%**		
Misoprostol action (h)	10.9 ± 4.9	8.4–13.4	10.9 ± 2.3	7.2–14.6	0.985
Time induction to delivery (h)	17.3 ± 4.8	14.8–19.7	15.2 ± 4.6	7.9–22.5	0.453
Initial Bishop score	2.1 ± 1.5	1.3–2.9	2.3 ± 0.5	1.5–3.1	0.869
1-min Appgar score	8.2 ± 1.1	7.6–8.7	7.8 ± 1.3	5.8–9.8	0.496
5-min Appgar score	9.0 ± 0.7	8.6–9.4	8.5 ± 0.6	7.6–9.4	0.207
Weight (grams)	3252.9 ± 271.8	3113.2–3392.7	3275.0 ± 450.0	2558.9–3991.1	0.898
Gestational age (weeks, mean ± SD)	39.6 ± 1.3	38.9–40.3	40.0 ± 0.7	38.8–41.3	0.578
**Spontaneous births (*n* = 42)**	***n* = 33, 78.6%**		***n* = 9, 21.4%**		
Misoprostol action (h)	10.8 ± 5.9	8.7–12.9	15.7 ± 5.5	11.5–20.0	0.029
Time induction to delivery (h)	16.3 ± 6.2	14.1–18.5	23.7 ± 7.7	17.8–29.6	0.004
Initial Bishop score	1.5 ± 1.3	1.0–2.0	2.6 ± 0.9	1.9–3.2	0.028
1-min Appgar score	8.6 ± 0.7	8.4–8.9	8.6 ± 0.7	8.0–9.1	0.851
5-min Appgar score	9.0 ± 0.5	8.9–9.2	9.3 ± 0.9	8.7–10.0	0.165
Weight (grams)	3472.7 ± 400.4	3330.8–3614.7	3316.7 ± 394.5	3013.4–3619.9	0.305
Gestational age (weeks, mean ± SD)	40.2 ± 1.2	39.7–40.6	39.9 ± 0.9	39.1–40.6	0.500
**Instrumental delivery (*n* = 11)**	***n* = 7, 63.6%**		***n* = 4, 36.4%**		*p*-value *
Misoprostol action (h)	13.8 ± 4.0	10.1–17.5	6.4 ± 1.7	3.8–9.0	0.007
Time induction to delivery (h)	17.9 ± 3.7	16.5–23.3	11.9 ± 2.9	5.3–14.4	0.001
Initial Bishop score	1.9 ± 1.1	0.9–2.9	1.5 ± 0.6	0.6–2.4	0.557
1-min Appgar score	8.6 ± 1.1	7.5–9.6	9.0 ± 0.8	7.7–10.3	0.527
5-min Appgar score	9.0 ± 0.8	8.2–9.8	9.5 ± 0.6	8.6–10.4	0.312
Weight (grams)	3385.7 ± 467.0	2953.8–3817.6	2887.5 ± 458.9	2157.2–3617.8	0.121
Gestational age (weeks, mean ± SD)	39.2 ± 1.5	37.7–40.6	39.6 ± 1.1	37.8–41.4	0.638

* *p* < 0.05 considered statistically significant.

## Abbreviations

BMI	Body mass index
*C*-section	Cesarean section
EU	European Union
FDA	Food Drug Administration
GPCR	G protein-coupled receptors
HELLP	Hemolysis, elevated liver enzymes, low platelet count.
HRPO	High risk pregnant obese women
IOL	Induction of labor—artificially initiated labor
MVI	Misoprostol vaginal insert
NICU	Neonatal intensive care unit
NSAIDs	Non-steroidal anti-inflammatory drugs
PROM	Prelabour rupture of membrane
P.G.s	Prostaglandins
PGE2	Prostaglandin E2
WHO	World Health Organization
SPSS^®^	Statistical Package for the Social Sciences, IBM^®^
T_max_	Time to peak plasma levels
